# Supported Decision‐Making Interventions in Mental Healthcare: A Systematic Review of Evidence on the Outcomes for People With Mental Ill Health

**DOI:** 10.1111/hex.70134

**Published:** 2024-12-22

**Authors:** Cathy J. Francis, Michael Hazelton, Rhonda L. Wilson

**Affiliations:** ^1^ Department of Nursing, School of Health and Biomedical Sciences RMIT University Bundoora Victoria Australia; ^2^ Central Coast Local Health District, NSW Health New South Wales Australia

**Keywords:** mental health, person‐centred care, shared decision‐making, supported decision‐making, UNCRPD

## Abstract

**Background:**

Most people with mental ill health want to be involved in decision‐making about their care, many mental health professionals now recognise the importance of this (at least *in‐principle*) and the Convention on the Rights of Persons with Disabilities enshrines the ethical imperative to support people in making their own treatment decisions. Nonetheless, there are widespread reports of people with mental ill health being excluded from decision‐making about their treatment *in practice*.

**Objectives:**

We conducted a systematic review of quantitative, qualitative and mixed method research on interventions to improve opportunities for the involvement of mental healthcare service users in treatment planning. We sought to consolidate and understand the evidence on the outcomes of shared and supported decision‐making for people with mental ill health.

**Methods:**

Seven databases were searched and 5137 articles were screened. Articles were included if they reported on an intervention for adult service users, were published between 2008 and October 2023 and were in English. Evidence in the 140 included articles was synthesised according to the JBI guidance on Mixed Methods Systematic Reviews.

**Results:**

There was evidence relating to the effects of these interventions on a range of outcomes for people with mental ill health, including on: suicidal crisis, symptoms, recovery, hospital admissions, treatment engagement and on the use of coercion by health professionals. There is favourable evidence for these types of interventions in improving some outcomes for people with mental ill health, more so than treatment‐as‐usual. For other outcomes, the evidence is preliminary but promising. Some areas for caution are also identified.

**Conclusions:**

The review indicates that when the involvement of people with mental ill health in treatment planning is supported, there can be improved outcomes for their health and care. Areas for future research are highlighted.

**Patient or Public Contribution:**

This systematic review has been guided at all stages by a researcher with experience of mental health service use, who does not wish to be identified at this point in time. The findings may inform organisations, researchers and practitioners on the benefits of implementing supported decision‐making, for the greater involvement of people with mental ill health in their healthcare.

## 1 Introduction

Most people with mental ill health want to be involved in decision‐making about their care and treatment [e.g. 1, 2, 3, 4, 5]. It is possible with the right support when required, including for those who are very unwell in acute hospital settings [e.g. 6, 7, 8]. At the same time, many mental health professionals now recognise the importance of shared or supported decision‐making in mental health treatment planning, at least *in‐principle* [e.g. 3, 4, 9, 10, 11, 12, 13, 14, 15, 16]. Furthermore, in 2008 the United Nations Convention on the Rights of Persons with Disabilities came into force and was subsequently ratified by many countries worldwide [17], enshrining the ethical imperative to support people in making their own treatment decisions, including for people with mental ill health [17].

Despite these factors, reports of people with mental ill health being denied a role in decision‐making about their treatment *in practice* are common. For example, in Australia, the recent Royal Commission into one State's Mental Health System, acknowledged that supported decision‐making is not routine and found service users regularly experience a lack of dignity, respect and choice, being excluded from decision‐making about their own care and treatment [18]. There are similar reports in other Australian jurisdictions [e.g. 19, 20, 21, 22]. and comparable scenarios reported in the United Kingdom [e.g. 23, 24, 25].

As described in detail in [26], there are numerous barriers to shared and supported decision‐making in mental health treatment planning in practice. One of the key reasons identified, is that clinicians remain sceptical of the associated benefits and outcomes of this type of decision‐making [e.g. 27, 28, 29, 30], adding to their hesitancy to facilitate it.

In this systematic review, the quantitative, qualitative and mixed method research into interventions that facilitate the greater involvement of mental healthcare service users in treatment planning is explored. The review was guided by the following questions: (i) what research has been undertaken in this field since 2008 when the UN CRPD came into force, (ii) what are the barriers to the implementation of these types of interventions, **(iii) what is the evidence on the associated outcomes for people with mental illness** and (iv) how do service users and clinicians experience it? While the systematic review integrates evidence to respond to all four questions, the overall results are outlined across several articles. In the first article, we focused on the findings to questions (i) and (ii) [26]. In this second article, we focus on the findings to **question (iii)**.

## 2 Methods

The methods for this review are outlined in detail in [26]. A brief summary is provided here. The PRISMA 2020 Checklist [31] was used to guide the review and the review protocol was registered with PROSPERO (CRD42022340117). A search string was designed and is available in full in [26] along with detailed inclusion and exclusion criteria.

Facilitated treatment planning was considered to include processes or practices where a specified person or digital tool assisted greater service user involvement in their treatment planning. Both shared and supported decision‐making interventions were included – definitions of each, and the relevance to this review are detailed in [26].

Databases were searched on 16–17 June 2021 and included CINAHL, Cochrane, Google Scholar, Medline, PsychInfo, PubMed and Scopus. An updated search of the same databases with the same search string was undertaken on 9 October 2023.

The included articles were reviewed according to the JBI Manual for Evidence Synthesis guidance on Mixed Methods Systematic Reviews [32], using the ‘convergent segregated approach’. According to the convergent segregated approach, the quantitative and qualitative data arising from the articles included in the review are of equal importance; they are synthesised independently in the first instance, then subsequently integrated into a ‘coherent whole’. Quantitative data were synthesised using a narrative summary as meta‐analysis was not possible. The findings of the review are presented across several papers, of which this is the second.

## 3 Results

A total of 140 articles were included in this review. A PRISMA flow chart, along with an overview of the research and its quality, is described in [26]. Across the 140 articles, the outcomes of facilitated treatment planning interventions for people with mental illness were investigated under the following categories:

- self‐harm and suicide,
- symptoms,
- recovery,
- hospital admissions,
- coercion (exposure to), and
- treatment engagement.

### 3.1 Self‐Harm and Suicide

Nine studies (12 articles) quantitatively researched the impact of a variety of facilitated treatment planning interventions on suicide‐related outcomes. In 3 of those studies (4 articles), **suicidal ideation** was investigated and was shown to be significantly reduced for participants, after the intervention compared to before [33, 34], and in comparison to a treatment‐as‐usual control group [35]. Bryan et al. [36] also report that more frequent use and better recall of enhanced crisis response plans were associated with significant reductions in suicidal ideation compared to treatment‐as‐usual.

Three (3) studies investigated **self‐harm** outcomes. One (1) study reported an improvement (significance not tested) in the length of time to first re‐presentation for self‐harm in an intervention compared to a control group [37]; the same study and another one reported no significant difference in self‐harming (number, proportion and/or frequency) between intervention and control groups [37, 38] – although Borschmann et al. [38] noted that their study was under‐recruited and therefore underpowered to detect significant differences. A study by Pearce et al. [39] compared Joint Crisis Planning with another treatment intervention (Democratic Therapeutic Community [DTC]). While the frequency of self‐harm acts was not significantly different between the two groups during follow‐up, the extent of self‐harm (measured by the Modified Overt Aggression Scale) was lower in both groups at 24‐month follow‐up compared to baseline, but significantly more so in the DTC group.

Results related to **suicide attempts** were also not definitive. One (1) study reported significantly reduced suicide attempts after the intervention compared to before [40]. When compared with a treatment‐as‐usual control group though – one study reported reduced (not significant) suicide attempts by participants in the intervention group [35], while another study indicated no significant difference [37]. In addition, 2 studies [41, 42] reported that the quality and completeness of a facilitated plan did not predict and was not associated with subsequent suicide attempts by participants. However, one of these studies [42] reported that their results suggested with a larger sample size, statistically significant findings would have been observed – that is, when the plans were more complete and certain parts of them were higher quality, this predicted a decrease in likelihood of future suicidal behaviour.

For other associated outcomes, improvements and in some cases, significant improvements, were reported for ‘*suicidal drivers*’ [33], *positive and negative emotional states* [43], *suicide‐related coping* and *suicide resilience* [34], and a ‘*clinically significant change*’ measure [33].

In summary, there is positive preliminary quantitative evidence suggesting facilitated treatment planning may reduce suicidal ideation and improve some of the emotional drivers that contribute to suicidal crisis, potentially more so than treatment as usual. On the impact of these interventions on self‐harming and suicide attempts, some promise is indicated for facilitated treatment planning to help reduce these outcomes, although results are mixed and further investigation is required to confirm whether there is added benefit to treatment‐as‐usual.

Only 2 of the studies [37, 44] collected qualitative data that reported on some relevant benefits from collaboratively prepared safety plan use. These included short‐term benefits such as helping with suicidal thoughts and long‐term benefits such as improved warning sign awareness and self‐management strategies.

Overall, it appears that facilitated treatment planning can contribute to helping people experiencing suicidal crisis and for some outcomes may potentially be more effective than treatment‐as‐usual. However, only a small number of studies have investigated any one aspect of suicidal crisis, and further research is required to strengthen evidence. Future mixed method research should as a minimum, consider incorporating measurements of all three aspects of suicidal crisis discussed above and seek to better understand people's experiences of facilitated treatment planning in relation to how it might potentially assist with suicide‐related crises.

### 3.2 Symptoms

The impact of facilitated treatment planning on various symptoms of mental ill health was approached by researchers in two different ways. Some studies investigated the effects on symptoms related to particular diagnoses, such as depression, while others studied the effects on psychiatric symptoms more generally using non‐diagnosis‐specific measures.

#### 3.2.1 Depression

Depression‐related symptoms were investigated by 9 studies (10 articles). Three studies indicated significant improvements after a facilitated treatment planning intervention compared to before [33, 45, 46], while six studies indicated no significant difference when interventions were compared to controls (treatment as usual) [38, 47, 48, 49, 50, 51, 52]. Of note, three of the later studies [47, 48, 50] reported that both intervention and control groups showed improved (including clinically significant improvement in one study) depression symptoms, although the groups were not significantly different. Furthermore, three (of six) of these RCTs noted limitations that may have affected the results and analyses [47, 48, 51, 52]. In the nine studies, 9 different interventions were investigated, using 7 different measures of depression symptoms and a range of follow‐up periods.

#### 3.2.2 Anxiety

The effects of facilitated treatment planning on anxiety was investigated by 2 studies (3 articles), both randomised controlled trials with 6‐12 months follow‐up periods. Both studies reported no significant difference between intervention and control groups [38, 51, 52] – these two studies used the same measure of anxiety symptoms (the anxiety subscale of the Hospital Anxiety and Depression Scale). The study by Lovell et al. [51, 52] reported problems with implementation in the intervention group, which may have impacted results.

#### 3.2.3 Schizophrenia

One study [53] has investigated symptoms specifically related to schizophrenia and it reported no significant difference between facilitated treatment planning intervention and control groups. Although, again, this study acknowledged some significant limitations with its implementation that may have impacted the findings (small sample size, trial not fully powered and the intervention was not always implemented as planned).

#### 3.2.4 Substance Misuse

Different aspects of substance misuse were investigated in two RCTs. Alcohol Dependence [54] and Drug Use [55] both significantly improved in intervention groups in comparison to control groups. Improvements were recorded in the intervention groups for Substance Misuse [54], Cannabis Dependence [54], Days of Primary Substance Use [55] and Addiction Severity [55]; however, the results were not significantly different to those in the control group. Joosten et al. [55] also reported no significant difference in Substance Dependence in the intervention compared to the control group.

#### 3.2.5 Psychiatric Symptoms (Consolidated)

There were 13 studies that investigated the impact of facilitated treatment planning interventions on psychiatric symptoms as measured by the Brief Psychiatric Rating Scale (BPRS) [56, 57], the Health of the Nation Outcome Scale (HoNOS) [54, 58], the modified Colorado Symptom Index (CSI) [59, 60], or a variety of other self‐report and clinician‐rated scales including the: EuropASI [55], CGI [61], SQ‐48 [62], FARS [63] and GHQ scales/questionnaires [39], among others. The studies investigated mostly different intervention types over a range of follow‐up periods (2–24 months).

Eight (8) of the 13 studies reported significant improvements in psychiatric symptoms, in intervention compared to control groups [54, 55, 57, 59, 60, 62], or after the intervention compared to before [58, 61]. A further two studies reported improvements that were not significant over the study period [63, 64]. Two (2) studies reported no significant difference between intervention and control groups [56, 65]. Salyers et al. [64] and Johnson et al. [56] both reported problems with the implementation of their studies that may have impacted results. While the two studies by Metz et al. [62] and Metz et al. [65] both reported that when their intervention was better implemented it was associated with significantly less Decisional Conflict, which was significantly associated with better clinical outcomes (e.g. reduced symptoms) (medium effect size) [62]. There was one further study [39] in which a Joint Crisis Planning intervention was trialled against another treatment intervention (DTC), and no significant difference was found between these two interventions for participants ‘Mental Health Status’. It is also interesting to note that the McNeely et al. [61] study found that after their intervention, self‐ratings and clinician‐ratings of illness severity were more closely aligned, than before the intervention.

No qualitative research specifically investigated the impact of facilitated treatment planning on participant symptoms. However, one study briefly outlines that participants reported a reduction in anxiety and panic attacks as one of the perceived benefits of undertaking that intervention [66]. Another study of a digital tool intervention included a symptom tracker and reported that many service user participants described this as an important and helpful component that assisted them to feel more control and involved in their care [67]. While another study [68] reported that the facilitated crisis planning process can help reduce the sense of urgency for people during a crisis, when symptoms can be intense and disturbing.

In summary, there is favourable quantitative evidence that facilitated treatment planning can significantly improve symptomatology of mental illness, potentially more effectively than treatment‐as‐usual or other therapeutic interventions (such as DTC). However, the evidence in relation to specific diagnoses is less clear. The little qualitative evidence that is available to date finds that people with mental ill health can have an interest in better understanding and managing their symptoms and that this can be important to them in the care they receive.

### 3.3 Recovery

There is no consensus on how to quantitatively measure recovery across the included studies – under the umbrella concept of recovery, we found relevant outcome measures that could fall under thirteen different categories.

The best‐studied category was **Quality of Life**, with 9 studies (10 articles) quantitatively investigating this outcome measure [38, 47, 51, 52, 55, 59, 60, 61, 65, 69]. All other categories were less well studied, they included: **Self‐efficacy/management** (7 studies) [34, 45, 54, 56, 61, 70, 71]; **Functioning** (6 studies) [45, 57, 58, 63, 65, 72]; **Work** (6 studies) [57, 58, 60, 61, 63, 73]; **Recovery** (6 studies, 7 articles) [51, 52, 56, 59, 60, 66, 74]; **Attitudes** (5 studies) [46, 64, 69, 70, 75]; **Wellbeing** (5 studies, 6 articles) [38, 51, 52, 54, 58, 66]; **Social functioning** (5 studies) [38, 39, 56, 76, 77]; **Empowerment** (5 studies) [59, 60, 71, 78, 79]; **Knowledge** (3 studies, 4 articles) [46, 70, 80, 81]; **Independence/Control** (2 studies) [76, 82]; **Activation** (2 studies) [61, 79]; and **Sense of coherence** (1 study) [61].

For all but one of these categories (the exception being ‘Sense of Coherence’), there was some quantitative evidence that the facilitated treatment planning interventions studied may improve these various aspects of recovery (Table 1).

**Table 1:** Summary of results and references for each quantitatively measured recovery‐related category.

Recovery measure	Facilitated treatment planning (FTP) superior to Control	FTP not significantly different to Control	Significant improvement after FTP intervention compared to before (no Control)	No significant difference before vs. after FTP intervention (no Control)
**Quality of Life**	**1 study** [60]	**6 studies** (7 articles) [38, 47*, 51*, 52*, 55, 59, 65] Note [47, 55]: – improved but not significantly different	**1 study** [69]	**1 study** [61] Note: reported greater improvements in QoL for clients that had higher levels of baseline motivational factors
**Self‐efficacy/management**	—	**2 studies** [54, 56] Note [54]: – improved but not significantly different [56] – control was unfacilitated treatment planning intervention	**4 studies** [34, 45, 61, 70] **Also relevant** [71] – 71% of participants reported that completing the JCP improved how much control they felt about their MH problem (however, a comparison of before vs. after was not undertaken and significance therefore not tested)	—
**Functioning**	—	**2 studies** [65, 72*]	**3 studies** [45, 57, 58]	**1 study** [63] Note: improved but not significantly different
**Work**	—	**2 studies** [60, 73] Note [60]: – improved but not significantly different	**2 studies** [57, 61] **Also relevant** [58]: – improvement, however, significance not tested	**1 study** [63] Note: significance not tested
**Recovery**	**3 studies** [59, 60, 74] Note [74]: Control was crisis card intervention	**2 studies** (3 articles) [51*, 52*, 56] Note [56]: Control was unfacilitated treatment planning intervention	**1 study** [66] Note: improvement but significance not tested	—
**Attitudes**	**1 study** [75] (Enhanced‐CRP intervention)	**1 study** [75] (Standard‐CRP intervention)	**4 studies** [46, 64, 69, 70]	**2 studies** [64, 70]
**Wellbeing**	**1 study** [54]	**2 studies** (3 articles) [38, 51*, 52*]	**1 study** [58] **Also relevant** [66]: – improvement but significance not tested	—
**Social Functioning**	**1 study** [76]	**3 studies** [38, 39, 56] Note [56]: – improved but not significantly different [39] – Control was another intervention (DTC)	—	**1 study** [77] Note: improved but not significantly different
**Empowerment**	**2 studies** [59, 60]	**1 study** [79]* Note: improved but not significantly different	**1 study** [78] **Also relevant** [71]: – 67% of participants reported that completing the JCP improved how they felt about themselves (however, a comparison of before vs. after was not undertaken and significance therefore not tested)	—
**Knowledge**	**1 study** (2 articles) [80, 81]	—	**2 studies** [46, 70]	—
**Independence/Control**	**1 study** [76]	—	**1 study** [82] Note: improvement but significance not tested	—
**Activation**	**1 study** [79*]	—	—	**1 study** [61]
**Sense of Coherence**	—	—	—	**1 study** [61]

*Significant limitations reported by authors with implementation of the study that may have impacted on analyses and results (e.g. problems with implementation, statistical tests were under‐powered, participants not randomised).

For most of the outcomes, there were at least some results indicating that the interventions did not add benefit over‐and‐above treatment‐as‐usual (TAU) controls (Table 1). However, for Quality of Life (QoL), Attitudes, Recovery, Wellbeing, Social Functioning, Empowerment, Knowledge, Independence/Control and Activation – there was also some evidence that facilitated treatment planning interventions can produce superior results to TAU controls (Table 1). In addition, there were three studies that compared facilitated treatment planning to another intervention – one reported a measure of recovery was significantly more improved in the facilitated treatment planning intervention compared to a crisis card intervention [74], while two studies reported that the interventions performed equally as well (e.g. no significant difference) when compared to an unfacilitated treatment plan and DTC for self‐efficacy/management and social functioning measures, respectively [39, 56] (Table 1).

There was a wide range of interventions investigated, measures used and follow‐up periods studied. This is likely to explain some of the variation in results.

The results from the included studies need to be considered in the context of some negative results that were reported in the qualitative literature, including one person in a study who reported feeling less independent after taking part in the intervention (to develop an Advance Agreement), because they came to realise how much power professionals have over hospital admissions [82]. In another study of a culturally‐adapted intervention for Hispanic service users, although the intervention decreased other types of stigma for people with mental ill health, it did result in increased negative perceptions about stigma related to anti‐depressant medication use [46]. In the study by Kayman et al. [44], some participants reported that discussing the topic of warning signs (as part of a collaborative safety planning intervention) was not helpful as it prompted urges toward self‐harm. While in Rogers and Dunne [83] some service users did not understand what recovery could mean for them, as it was not explained by healthcare professionals in the intervention.

Overall, there were more qualitative data relating to recovery and its various elements than that available for the other outcomes considered in this article. Qualitatively, service users spoke often about improvements in their **understanding, awareness, learning and insights** on their illness and its management, and the importance of those factors to their recovery [37, 61, 66, 67, 68, 70, 81, 82, 84, 85, 86, 87, 88]. Service users also spoke frequently about the following benefits of facilitated treatment planning interventions: improvements in feelings of **empowerment** (including self‐esteem, confidence, dignity and respect) [27, 61, 66, 70, 77, 89, 90, 91, 92, 93, 94, 95]; improvements in being able to **manage their illness themselves** [37, 61, 66, 70, 84, 85, 86, 91, 96]; increased **feelings of hope, optimism, responsibility and ownership for wellness** [34, 61, 67, 70, 81, 92, 93, 96, 97, 98]; and about having more **control** over their illness, recovery, healthcare and wellbeing [38, 67, 68, 82, 89, 90, 94, 95, 99], noting though that one study also described no change in feelings of control for some participants [82].

In the context of their recovery, service users spoke less frequently about improvements in their **relationships, support networks and social activities** [67, 70, 96], and to their **motivation and participation** in care [61, 82, 84, 94]. On crisis plans specifically, service users found they **lessened concerns about a future crisis** occurring [68, 99]; two studies described improvements in the sense of **wellbeing** for participants [77, 93] and one study described a participant that had improved their **work** situation [96].

Clinician perspectives were less studied in the qualitative literature. However, when studied, clinicians most often mentioned that the facilitated treatment planning interventions were useful in increasing patient **learning and understanding** about their illness and recovery journey [84, 100, 101, 102] as well as improvements in patient **motivation and involvement** [82, 96, 100, 102, 103]. Clinicians also mentioned: patients **feelings of hope and responsibility/ownership** for their recovery process [100, 101, 102, 103], the benefit of being able to show patients their **strengths and progress** [100] and in patients feelings of greater **independence or control** [82, 104], respectively.

According to descriptions in service user qualitative data, a range of factors would seem to feature in people's recovery processes. Many of these factors align well with the recovery categories that were investigated quantitatively. For example service users most frequently spoke, in qualitative data, about improvements in their **understanding, awareness, learning and insights** – this would align well with the quantitative outcome category of **Knowledge**. Likewise, a person being able to better **manage their illness themselves** aligns well with the quantitative outcome category of **Self‐efficacy/management**. While increased **feelings of hope, optimism, responsibility and ownership for wellness** align well with the quantitative outcome measure of **Attitudes**. And so on. Similarly, for clinician perspectives.

Notably, some of the factors most often mentioned by service users in qualitative data (i.e. improved **Knowledge, Empowerment, Control**), were amongst the least studied in the quantitative data. Clinicians too focused on improved patient **Knowledge** but also on **Activation** as a benefit of the interventions to people's recovery in qualitative data, however activation was one of the least mentioned benefits for service users and was little studied in the quantitative data.

**Quality of Life** was not specifically mentioned by any service users or clinicians in qualitative data collected as relevant to or indicative of recovery (this was also noticed by Shillington et al. [88] in their study). Quality of Life measures may be most useful in discussions of recovery when considered alongside other measures that are more directly meaningful to service users.

To date there is favourable evidence, both quantitative and qualitative, that facilitated treatment planning interventions can help with people's recovery from mental ill health. For numerous aspects of recovery, this type of intervention may potentially be more effective than treatment as usual or other interventions. Going forward, replication of studies on existing interventions to strengthen evidence is needed. Future research would also benefit from a consolidation of the measures investigated and instruments utilised under the umbrella of recovery‐relevant outcomes ‐ this would be best guided by what service users feel is most important to them in the context of recovery. Indicatively, based on the findings of this review: **Knowledge**, **Empowerment**, **Self‐efficacy** and **Independence/Control** are elements of recovery that are important to investigate further. Research also needs to take account of the potential for negative impacts of facilitated treatment planning on people's recovery.

### 3.4 Hospital Admissions

Numerous studies considered the effects of facilitated treatment planning on various quantitative admissions‐related measures (Table 2), including:

- **Hospital Admissions** – 17 studies,
- **Involuntary Admissions** – 7 studies (8 articles),
- **Admission Duration** – 13 studies,
- **Time to Re‐admission** – 4 studies, and
- **Likelihood of Re‐/admission** – 3 studies.

**Table 2:** Summary of results and references for each quantitatively measured hospital admission‐related measure.

Admissions measure	Facilitated Treatment planning (FTP) superior to Control	FTP not significantly different to Control	Significant improvement after FTP intervention compared to before (no Control)	No significant difference after vs. before FTP intervention (no Control)
**Hospital admissions**	**2 studies** [43, 56*] Note [43]: – for enhanced CRP [56] – Control was unfacilitated treatment planning	**9 studies** [27*, 37, 39, 59, 60, 73, 74*, 105*, 1^06^] Note [105]: – improved but not significantly different [39] – Control was DTC [74] – Control was crisis card	**2 studies** [61, 107] **Also relevant** [77]**:** – improved, however, significance not tested/indicated [41] – individuals with higher quality plans were significantly less likely to be hospitalised in follow‐up period	**1 study** [108] Note: improved, however, not significantly different (except for patients with bipolar disorder, for which there was a significant difference) **Also relevant** [42]**:** – safety plan quality and completion did not predict psychiatric hospitalisation
**Involuntary admissions**	**3 studies** [59, 60, 105*] Also relevant [109]: – Odds of experiencing coercion (including involuntary admission) significantly lower in intervention vs. control	**2 studies** (3 articles) [27*, 74*, 110] Note [74]: – Control was crisis card	**1 study** [107]	—
**Duration of admission**	**2 studies** [35, 60]	**7 studies** [27*, 39, 56*, 72*–74*, 105*] Note [39, 56, 74]: – Controls were different treatment interventions not TAU	**4 studies** [57, 61, 107, 108]	—
**Time to re‐admission**	**1 study** [56] (Control was unfacilitated treatment planning)	**3 studies** [37, 73, 74*] Note [74]: – Control was crisis card	—	—
**Likelihood of re‐admission**	**1 study** [43] (with enhanced CRP – clinicians significantly less likely to recommend hospitalisation)	**1 study** [73]	**1 study** [108] (risk of re‐admission 58% less after intervention – significant for participants with schizophrenia, but not bipolar disorder)	—

*Significant limitations reported by authors with implementation of the study that may have impacted on analyses and results (e.g. problems with implementation, statistical tests were under‐powered, participants not randomised).

The impact of facilitated treatment planning on **hospital admissions** and **admission duration** was relatively well studied, with a wide range of intervention types, methods and follow‐up periods investigated. Results suggest that facilitated treatment planning can assist in reducing hospital admissions and admission duration for mental ill health (Table 2). Although, most of the studies indicated that facilitated treatment planning is not significantly different to TAU controls (Table 2) – suggesting that it may not add benefit (in terms of these measures) over‐and‐above TAU. However, there was also evidence that some interventions were actually superior to TAU for reducing hospital admissions [43] and admission duration [35, 60] (Table 2). In addition, three studies compared facilitated treatment planning to other therapeutic interventions (DTC, crisis card, unfacilitated treatment planning) and in all three it performed at least as well as, in one case, better than, the other interventions (Table 2). Gamarra et al. [41] report that individuals with higher‐quality plans were significantly less likely to be hospitalised, while Green et al. [42] indicated that facilitated safety plan quality and completeness did not predict hospitalisation.

On **involuntary hospitalisation**, there is favourable evidence in the included articles that facilitated treatment planning interventions reduce the use of this restrictive practice. In this review, there are three recent RCTs that indicate it can be more effective at reducing involuntary treatment than TAU, with one earlier study indicating no significant difference between intervention (JCP + TAU) and control (TAU) groups (Table 2). All of the studies measuring involuntary admissions were researching the same general type of intervention, that is facilitated crisis planning (JCP, PAD, Advance Directive). Together, this evidence builds on that in existing meta‐analyses, which have shown these types of interventions can significantly reduce the risk of involuntary hospital admissions compared with other intervention types [111, 112]. Interestingly, when German psychiatrists were asked if they thought joint crisis plans reduced involuntary admissions and compulsory treatment, 23% said yes, 44% were not sure and 27% said no [29].

**Time to re‐admission** and **likelihood of re‐admission** were less well studied – there is some evidence that facilitated treatment planning interventions may not perform any better than TAU controls [37, 73], but also some evidence that they can be better than TAU [43] and may perform at least as well or better than other therapeutic interventions for these two outcomes [56, 74] (Table 2).

It is also noted that for a number of the studies relevant to this section, limitations were identified including problems with the implementation of the intervention, or with statistical power due to small sample sizes, which may have affected results and analyses [e.g. 27, 37, 56, 72, 74, 105].

Although not specifically a focus of qualitative research, there are a few reports of both service user and professional views that facilitated treatment planning can reduce or avoid hospitalisation [68, 84, 89, 99, 102], for example:

Patient: ‘It prevents me from being hospitalised’. [68]

[Staff participant:] if you're treated well, you simply feel better, and if you're able to say what you need and get help with that, then the hospital stay should reasonably become shorter. [102]

In summary, results indicate some potential for facilitated treatment planning interventions to have positive effects that can reduce the number, type, length and likelihood of hospital admissions – particularly for reducing involuntary admissions when facilitated crisis (or advance) planning is undertaken.

### 3.5 Coercion (Exposure to)

In addition to involuntary hospitalisation which has been covered in Section 3.4, our review identified 6 studies that quantitatively investigated the impact of facilitated treatment planning on the use of coercion in mental health service provision [27, 38, 74, 107, 109, 113].

The definition of coercion differed across these studies. Two considered the impacts of facilitated treatment planning on perceived coercion [27, 38], two considered coercion in terms of the use of seclusion [107, 113] while others considered coercion more broadly – for example to include seclusion, restraint or forced medication [74] or to additionally include transport by police, use of handcuffs or involuntary commitment [109]. The studies in this section investigated mostly facilitated crisis plans (Joint Crisis Plans [27, 38, 74], Advance Directive [107], PAD [109]) as well as a Care Plan [113].

Of note, in the study by Swanson et al. [109], rates of coercion were much higher for people who reported episodes of incapacity during mental health crisis. That is, when people were at their most vulnerable and most in need of care, they were more often exposed to being transported by police, hand‐cuffed, locked in seclusion, physically restrained and/or forced medication.

Overall though, Swanson et al. [109] reported a significant reduction in coercive measures experienced by people with a facilitated PAD compared to those without one at 6 months follow‐up, and in the odds of experiencing any coercive measure during 24 months follow‐up. The same study also reported that the mean and cumulative rate for the measure of coercion used were both reduced (not significantly) for those with a PAD over 24 months. Another study reported a reduction (not significant) in the number of days people spent in a locked room, after a facilitated treatment planning intervention compared to before [107].

Two studies must be highlighted in this section. First, the findings of Elzubeir and Dye [113] who reported that seclusion was used 3 times *less often* after a facilitated planning intervention was implemented in a Psychiatric Intensive Care Unit. However, when used, it was for a much *longer duration* in a less restrictive seclusion setting (statistical significance not analysed). A similar trend was reported in the study by Rixe et al. [74] which compared facilitated treatment planning (JCP) with another treatment intervention (crisis cards). In their study, JCP resulted in fewer overall coercive measures (forced medication, physical restraint, isolation) and shorter duration of physical restraint – but a higher number and cumulative duration of isolations (although not statistically significant). Both of these studies suggest that it can be possible for a shift to occur as a result of interventions, from the use of one form of coercion to another by mental health service providers.

Two studies of a JCP intervention found no significant differences in perceived coercion (for service users) in the intervention compared to control groups [27, 38].

Thornicroft et al. [27] reported problems with implementation of the intervention that may have influenced the findings, while Rixe et al. [74] reported high dropout rates that may have lowered the statistical power to detect significant effects.

Again, in the studies included in this review, there is very little qualitative data available on the impacts of interventions on exposure of people with mental ill health to coercion use by service providers. In one study, staff who were interviewed noted a decrease in their use of involuntary procedures on wards participating in the intervention, linked with greater staff tolerance, use of dialogue and flexibility in routines [102]. Clark et al. [114] also outlined some encouraging reports by nurses in a study where facilitated treatment planning was implemented. For example:

Rather than say “off you go to seclusion” or use seclusion as a threat, it doesn't cross my mind now (since the introduction of PBSPs). I kind of engage with the patients more, become more vigilant and spent time with my patients. [P9] [Nurse] [114]

Swanson et al. [109] document one narrative interview with a service user, who felt that having prepared a (facilitated) PAD was useful in reducing coercion:

**Excerpt**: “[In the hospital] we talked about what was in the PAD… I did not receive any treatments that I did not want. They were very respectful … I really felt like the hospital took better care of me because I had my PAD….” [109]

However, Henderson et al. [71] report that in an audit of participant experiences for those with a JCP who were hospitalised in that study, 4 (of 13) experienced the use of coercive measures (e.g. involuntary admission or treatment) against explicit preferences outlined in their plans.

Participants in another study investigating the use of Advance Agreements, thought having independent oversight of plan quality and making the plans mandatory practice, could reduce the sense of coercion felt by service users [82]. It was not clear if these views were of service users, clinicians or both.

On balance, the results indicate some promise for facilitated treatment planning interventions to potentially reduce the use of coercion in mental health service provision. However, caution is recommended – future studies must ensure that any shift in the nature or focus of coercion, from one type to another or from some individuals to others is detected and understood, so that it can be avoided and prevented in future.

In relation to the use of coercion and the preparation of facilitated treatment plans to prevent its use on people with mental ill health, Clark et al. [114] also reports that mental health professional's perceptions of whether service users were ‘responsible’ for their behaviour or not, could influence both the preparation and implementation of plans by clinicians and their subsequent use of coercion. Future research could investigate the role of facilitated treatment planning in shaping and changing mental health professionals' views on their use of coercion.

### 3.6 Treatment Engagement

Included under this outcome are measures of appointment attendance, engagement with services and medication use.

Treatment engagement is one of the better quantitatively studied outcomes (22 studies, 24 articles). However, what has been studied has been a range of: interventions (e.g. a variety of shared decision‐making interventions, crisis plans, care plans, safety plans and others); measures (e.g. the Service Engagement Scale SES17, drop‐outs and no‐shows, therapy initiation, outpatient/therapy attendance, a range of ways of measuring medication adherence) and follow‐up periods (1 month to 2 years).

Perhaps reflective of the variation in the research methods, quantitative results on treatment engagement are mixed. On the one hand, 5 studies reported that facilitated treatment planning interventions were **superior to** treatment‐as‐usual control groups in improving treatment engagement (including various measures of medication adherence, initiation of treatment, therapy adherence and engagement in outpatient services) [47, 50, 53, 115, 116]. Two more studies reported improved treatment initiation and retention in intervention compared to control groups, although significance (or not) of these findings was not indicated [79, 87]. And, a further 2 studies [72, 106] reported improved (although not significantly) treatment engagement in intervention compared to control groups. In addition, the study by Khazaal et al. [107] found that before their facilitated treatment planning intervention, all patients (100%) revealed non‐adherence or irregular observance to treatment. After the intervention, during the last 6 months (of a 2 year follow‐up period) only one patient (5.6%) revealed such non‐adherence or irregular observance. Relatedly, Wilder et al. [117] found significantly improved medication adherence if a person was prescribed at least one medication requested in their PAD; Sanchez et al. [46] reported that when depression knowledge increased, people were significantly more likely to engage in counselling and report taking medication; and Henderson et al. [71] found that immediately after their facilitated treatment planning intervention (JCP) 69% of patients perceived the likelihood that they would continue with care was improved (although by 15 months follow‐up this had reduced to 28%).

On the other hand 10 studies (12 articles) report that, in terms of various measures of treatment engagement, facilitated treatment planning interventions were **not significantly different** to treatment‐as‐usual control [27, 38, 48, 49, 50, 51, 52, 62, 106, 110, 118] or comparison [119] groups. In addition, Gamarra et al. [41] found no association between safety planning completeness or quality and the likelihood of a person attending four or more outpatient appointments. Both the CRIMSON study [27, 110] and the EQUIP study [51, 52] reported problems with implementation that may have impacted results.

A small number of included articles reported on relevant qualitative data collected, which represented a range of views on the influence of facilitated treatment planning interventions on treatment engagement, focused on medication use. Some service users and clinicians indicated that these interventions may contribute to increased treatment engagement through improved communication and knowledge [53, 67, 68, 77], for example:

It made me understand why I am taking what I am taking. [53]

it made her [the psychiatrist] see all the side effects and what they do. [53]

One study reported that a small number of people found that the intervention made no difference or that facilitated treatment planning could highlight the negative aspects of medication [53]. Future interventions should prepare both clinicians and service users for this possibility arising through the shared or supported decision‐making process, so that service users can be appropriately supported to make a decision that works best for them and their circumstances.

Together, the results from quantitative and qualitative studies at least indicate favourable evidence for some types of facilitated treatment planning interventions to have positive effects on treatment engagement and, in some cases, to potentially be superior to treatment‐as‐usual controls. Future studies need to replicate testing and investigation of existing interventions to confirm the evidence‐base. Greater consistency in the measures and follow‐up periods used in quantitative investigations would also assist in comparison and interpretation of results. Further qualitative research will build on understanding the reasons people have for engaging, or not, in treatment after undertaking facilitated treatment planning.

## 4 Discussion

Supported decision‐making is a process proposed to empower people with mental ill health and to promote the delivery of healthcare in line with human rights law and policy [120]. It has been noted that practical research is needed to inform all stakeholders, including individuals and health professionals, on the efficacy and scalability of supported decision‐making [120]. Indeed, we know from the research literature that health professionals, although generally supportive *in‐principle* of shared and supported decision‐making concepts, remain sceptical of the efficacy and effectiveness of such interventions for achieving improved practical outcomes in mental healthcare [26]. Therefore, the practical research highlighted above is not only needed but is crucial. Such research in mental healthcare has been in an expansion phase over the last couple of decades, with an increasing range of different interventions and outcome measures being explored. This review sought, among other things, to consolidate evidence available on the outcomes (or efficacy and effectiveness) of facilitated mental health treatment planning, including both shared and supported decision‐making, since 2008 when the CRPD came into force.

Our review indicates some important outcomes for people with mental ill health (Table 3). Specifically, there is favourable evidence for these types of interventions in reducing, more so than treatment‐as‐usual, suicidal ideation and drivers, and some symptom measures. Also, for improving some important aspects of recovery that people with mental illness appear to particularly value, including: knowledge, empowerment and independence/control. The review also adds to the growing body of evidence [e.g. 111, 112]. that shared and supported decision‐making in mental health treatment planning can importantly reduce the use of involuntary hospitalisation in mental health service provision. Furthermore, some promise is indicated for other meaningful outcomes for people with mental ill health, including potential reductions in likelihood and duration of hospitalisation, improvements in treatment engagement and reductions in the use of some types of coercion by health professionals. The results of the review also indicate that there is some need for caution in future research, to ensure that potential negative impacts of these types of interventions are understood and can be addressed.

**Table 3:** Summary of integrated quantitative and qualitative evidence on comparisons of facilitated treatment planning interventions to treatment‐as‐usual.

Outcomes for people with mental ill health	Evidence favouring superiority to TAU	Evidence equivocal	Evidence favouring no improvement to TAU	Caution noted
**Suicidal Crisis**				
Suicidal ideation	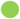			
Self‐harming		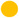		
Suicide attempt		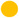		
Suicidal drivers	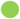			
**Symptoms**				
Depression			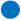	
Anxiety			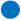	
Schizophrenia		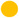		
Substance misuse	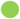			
Psychiatric symptoms (consolidated)	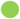			
**Recovery**				
Quality of Life			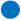	
Self‐efficacy/management		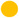		
Functioning			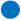	
Work			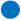	
Recovery	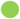			
Attitudes	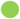			
Wellbeing		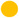		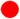
Social functioning		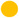		
Empowerment	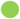			
Independence/control	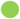			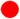
Patient activation	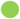			
Knowledge	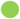			
Sense of coherence		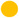		
**Hospital Admissions**				
Admissions			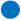	
Involuntary admissions	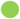			
Duration of admission		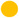		
Time to re‐admission		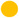		
Likelihood of re‐admission		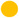		
**Physical Coercion (exposure to)**		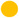		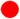
**Treatment Engagement**		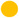		

*Note:* Green circle = evidence favouring superiority to TAU; Yellow circle = evidence equivocal; Blue circle = evidence favouring no improvement to TAU; Red circle = caution noted.

A key challenge for future research on shared and supported decision‐making in mental healthcare, will be the shift from the expansion phase into the integration and consolidation phases. This shift is necessary so that the evidence base for facilitated treatment planning can be strengthened and clarified, so that meta‐analyses can be performed, absolute benefits can be determined and our understanding of what influences effectiveness can be enhanced through qualitative research. Toward this, the *experiences* of those who have been participants in shared and supported decision‐making in mental health treatment planning interventions will be important.

## 5 Review Limitations

The limitations of this review have been previously outlined in [26].

## Author Contributions

**Cathy J. Francis:** conceptualisation, writing–original draft, methodology, validation, writing–review and editing, formal analysis, project administration, data curation. **Michael Hazelton:** writing–review and editing, project administration, supervision, validation. **Rhonda L. Wilson:** conceptualisation, methodology, validation, writing–review and editing, project administration, supervision.

## Conflicts of Interest

The authors declare no conflicts of interest.

## Data Availability

The data that support the findings of this study are available from the corresponding author [C.F.] upon reasonable request.
